# Sharing public health data and information across borders: lessons from Southeast Asia

**DOI:** 10.1186/s12992-018-0415-0

**Published:** 2018-09-29

**Authors:** Marco Liverani, Srey Teng, Minh Sat Le, Richard Coker

**Affiliations:** 1 Department of Global Health and Development, London School of Hygiene & Tropical Medicine, London, UK; 2 Faculty of Public Health, Mahidol University, Bangkok, Thailand; 3 Department of Communicable Disease Control, Ministry of Health, Phnom Penh, Cambodia; 4 Ministry of Science and Technology, Hanoi, Vietnam

**Keywords:** International health regulations, Infectious disease surveillance, Health data and information sharing, Southeast Asia, Health information systems, Regional health cooperation

## Abstract

**Background:**

The importance of data and information sharing for the prevention and control of infectious diseases has long been recognised. In recent years, public health emergencies such as avian influenza, drug-resistant malaria, and Ebola have brought renewed attention to the need for effective communication channels between health authorities, particularly in regional contexts where neighbouring countries share common health threats. However, little empirical research has been conducted to date to explore the range of factors that may affect the transfer, exchange, and use of public health data and expertise across borders, especially in developing contexts.

**Methods:**

To explore these issues, 60 interviews were conducted with domestic and international stakeholders in Cambodia and Vietnam, selected amongst those who were involved in regional public health programmes and networks. Data analysis was structured around three categories mapped across the dataset: (1) the nature of shared data and information; (2) the nature of communication channels; and (3) how information flow may be affected by the local, regional, and global system of rules and arrangements.

**Results:**

There has been a great intensification in the circulation of data, information, and expertise across borders in Southeast Asia. However, findings from this study document ways in which the movement of data and information from production sites to other places can be challenging due to different standards and practices, language barriers, different national structures and rules that govern the circulation of health information inside and outside countries, imbalances in capacities and power, and sustainability of financing arrangements.

**Conclusions:**

Our study highlights the complex socio-technical nature of data and information sharing, suggesting that best practices require significant involvement of an independent third-party brokering organisation or office, which can redress imbalances between country partners at different levels in the data sharing process, create meaningful communication channels and make the most of shared information and data sets.

## Background

It has long been recognised that “diseases know no borders” and have the potential to spread across vast geographic spaces, particularly in regions where similar epidemiological profiles, socio-economic drivers, and frequent movements of people or animal carriers facilitate disease transmission and persistence. The Ebola epidemic in West Africa (2013–2016) is a prime example of a regional health crisis, exacerbated by the mobility of infected patients across the porous borders of Guinea, Liberia, and Sierra Leone [1, 2]. Other examples of diseases which present a strong cross-border dimension include malaria and artemisinin-resistant malaria in Southeast Asia [3, 4], cholera in Sub-Saharan Africa [5], Zika in Latin America [6], avian influenza in Southeast Asia [7] and diseases that receive less global health attention such as onchocerciasis [8] and Japanese encephalitis [9]. In such contexts, the development of effective communication channels between health authorities and other stakeholders within and between countries is thought to be crucial to enable effective responses in emergency situations as well as routine surveillance of endemic diseases, sustain progress towards disease elimination, and inform policy development through the exchange of data, information and expertise [8, 10]. In recognition of this, the World Health Organization (WHO) and other global health actors have issued recommendations and guidelines to promote closer links between regional partners [11, 12]. Notably, the International Health Regulations (2005) encourage “bilateral or multilateral agreements or arrangements” in cross-border regions to enable collective action, including “the direct and rapid exchange of public health information between neighbouring territories of different States” (Article 57). In keeping with these recommendations, regional public health initiatives have been established in several parts of the world to facilitate data and information exchange. In Europe, disease surveillance networks have been in operation since the 1980s and are currently integrated and managed under The European Surveillance System (TESSy), a web-based technical platform which collects and disseminates national surveillance data from all European Union (EU) and European Economic Area (EEA) countries using standardized formats [13]. From the 1990s, bilateral and regional public health networks or programmes have also been established in low- and middle-income contexts, including in Africa [14], Southeast Asia [15–17], the Middle East [18, 19], and South America [20]. In addition to their public health value, these initiatives are an important illustration of the emergence and strengthening of South-South cooperation, understood as “a process whereby two or more developing countries pursue their individual or collective development through cooperative exchange of knowledge, skills, resources and technical expertise” [21].

Yet these processes have occurred amidst increasing awareness that international health cooperation is challenging. While good practices and outcomes have been reported [15, 19, 22], studies informed by political and institutional perspectives have identified constraints to the achievement of meaningful and effective cooperation, given the divisive force of state politics and interests, gaps in domestic capacities, and weaknesses in institutional frameworks to support collective action amongst regional partners [23–25]. In the public health literature, extensive lists of barriers have also been generated to map constraints to health data and information sharing within and across countries [26]. For example, van Panhuis and colleagues identified 20 potential barriers across technical, motivational, economic, political, legal and ethical domains [27]. However, as found in the same review, little empirical research has been conducted to date to explore these issues in a systematic way. Further, most contributions have been normative in nature or focused solely on institutional and policy developments, with scant integration of qualitative evidence from the field, especially in developing contexts. A number of qualitative studies of health data sharing have been conducted [28–30], but these have focused on researchers and research data, not on health officers, implementers and data from national health information systems.

Considering this gap in knowledge, we present and analyse here findings from an exploratory qualitative study of regional health cooperation in Southeast Asia, with a focus on issues and challenges that may influence health data and information sharing. In the wake of recent regional health emergencies, such as avian influenza H5N1, severe acute respiratory syndrome (SARS), and artemisinin-resistant malaria, several programmes have been developed in the region to share epidemiological data and expertise between national health authorities and practitioners, and develop an integrated approach to address common health problems. The collection of views and experiences from participants who have been involved in these initiatives provides a unique insight to better understand the practice of South-South health data sharing in a context characterized by imbalances between more and less resourced countries. After the description of methods, the result section summarises findings from interviews with key informants. Reflecting on research findings, the discussion elaborates on institutional and organisational arrangements that are likely to promote the establishment of effective and meaningful public health networks in Southeast Asia as in other contexts.

## Methods

### Study context

Regional health cooperation in Southeast Asia provides a suitable or “critical” case [31] to explore the complexity of challenges affecting health data and information sharing in LMICs, given gaps in capacities between more and less resourced countries, differences and commonalities between national health sectors, and the variety of regional programmes and networks implemented in recent years. Early developments date back to the 1990s, when the relaxation of international tensions in the region, combined with concerns about endemic and emerging infections which spread across and beyond regional borders, attracted considerable donor investments to support regional initiatives for disease prevention and control [32]. Since then, several networks and programmes have been established, characterised by different configurations, organisational arrangements, timeframes, and membership (Table 1). Some initiatives have been horizontal in scope; others have focused on specific diseases including malaria [33], HIV [34], and avian influenza [35]. In keeping with the global health agenda, and in recognition of the regional dimension of the problem, substantive efforts have been made to strengthen regional malaria initiatives, within the wider context of the Strategy for Malaria Elimination in the Greater Mekong Subregion (2015–2030) and the Emergency Response to Artemisinin Resistance [36]. The consolidation of organisations for political and/or economic cooperation in Southeast Asia has also provided institutional frameworks to support regional public health networks and programmes [37]. To different degrees, the prevention and control of infectious diseases has been on the agenda of the Association of Southeast Asian Nations (ASEAN), the Asian Development Bank (ADB), and the Asia-Pacific Economic Cooperation (APEC), in joint action with the WHO South East Asia (SEARO) and Western Pacific Regional Offices (WPRO). For example, between 2004 and 2008, the ASEAN Secretariat managed an Emerging Infectious Disease (EID) programme aimed to strengthen public health capacities regionally through joint training courses, seminars and workshops, links between human and animal health sectors, and exchanges of staff between laboratories in the region [37].

**Table 1:** Examples of regional public health programmes in Southeast Asia

	Timeframe	Health issue(s)	Activities	Main Funders	Coordination	Membership
ASEAN + 3 EID Programme	2004–2009	Infectious diseases (horizontal)	Surveillance, capacity building, research, policy analysis, training	AusAid	ASEAN Secretariat	Indonesia, Malaysia, the Philippines, Singapore, Thailand, Brunei, Myanmar, Cambodia, Laos, Vietnam, China, Japan, Korea.
Emergency Response to Artemisinin Resistance (ERAR) in the Greater Mekong Subregion	2013–2016	Malaria	Drug surveillance, health service delivery, capacity building, data and knowledge sharing	BMGF, DFAT	WHO (Country Office Cambodia)	Cambodia, China, Lao PDR, Myanmar, Thailand, Vietnam
Greater Mekong Subregion Communicable Disease Control Project (GMS-CDC)	2005-ongoing	Infectious diseases (horizontal)	Surveillance, capacity building, training, information sharing	Asian Development Bank	Regional Coordination Unit, Vientiane	Cambodia, Laos, Vietnam
Greater Mekong Subregion Malaria Control Project (CAP-Malaria)	2011–2016	Malaria	Training, health promotion, health service delivery, data and knowledge sharing	USAID	University Research Co.	Cambodia, Myanmar, Thailand
Lao PDR-Cambodia One Health Surveillance and Laboratory Network (LACANET)	2014-ongoing	Infectious diseases (horizontal)	Surveillance, data and knowledge sharing, capacity buidling (laboratory), research	European Union	Institut Pasteur	Cambodia, Lao PDR
Mekong Basin Disease Surveillance Project (MBDS)	1999-ongoing as a foundation	Infectious diseases (horizontal)	Surveillance, training, capacity building, information sharing	Rockefeller Foundation	MBDS Office, Bangkok	Cambodia, China, Laos, Myanmar, Thailand, Vietnam
Stop Transboundary Animal Disease & Zoonoses (STANDZ)	2011–2016	Animal diseases and zoonoses	Surveillance, preparedness, training, research, health promotion, data and knowledge sharing	Government of Australia (DFAT)	World Organisation for Animal Health (OIE)	Surveillance, preparedness, training, research, health promotion, data and knowledge sharing
Regional HIV/AIDS Data Hub	2010-ongoing	HIV	Institutional development, data sharing	ADB	ADB	–

The sharing of data, information, and expertise between country partners has been a key feature in most initiatives. Sub-regional networks such as the ADB-sponsored Greater Mekong Subregion Communicable Disease Control project (GMS-CDC) or the Mekong Basin Disease Surveillance (MBDS) network have appointed contact persons at selected border provinces, who are required to e-mail standard forms to their counterparts in the neighbouring country, including information on cumulative number of cases and deaths, outbreak location, and presumptive causes of disease outbreaks. In both networks, data sharing schedules are tailored to different epidemiological and risk profiles: outbreaks of emergency diseases such as H5N1, cholera and SARS must be communicated to the WHO and the neighbouring province within 24 h, while the data sharing schedule for endemic diseases such as malaria, TB, or HIV is more relaxed (i.e. monthly or quarterly). Data sharing activities have also been included in vertical programmes for disease control. From the 1990s, various efforts to build a regional malaria database have been made [38] and revived as part of the current strategy of malaria elimination in the GMS. Another example is the AIDS Data Hub, based in Bangkok, which collects and publishes HIV/AIDS regional data by scanning published literature and web sites, and receiving data from a network of country partners [39].

### Data collection and analysis

Data collection for this project primarily involved key informant interviews, conducted in Cambodia and Vietnam, between January and April 2016, to capture diversity and similarities in health and surveillance systems, while relying on strong professional links between the researchers and stakeholders in the two countries. Key informants were identified amongst those who were involved in active or recently completed regional programmes and data sharing activities (or could provide expert information and views), after preliminary consultations with local partners; additional participants were identified through snowball sampling. In order to account for different perspectives, we aimed to recruit participants from ministries of health and other government bodies (both at the central and provincial level), managers of vertical programmes and departments of communicable disease control, data management officers, and experts at international organisations. The researchers (ML, ST, SML) contacted individuals or offices by phone or email to request participation in the study. Prior to each interview, participants were provided an information sheet with details about the project aim and methods and a tentative list of topics to be covered during the meeting. Given the exploratory nature of this study, we prioritised a grounded approach [40], rather than using a predetermined analytical framework. Thus, the interview schedule was tailored to the role and expertise of informants and lightly structured to elicit views and experiences about their involvement in regional data and information sharing activities, both in the event of disease outbreak and for routine surveillance of endemic diseases. Further, participants were asked to comment on the value of data and information sharing and to discuss factors and challenges that may affect the transfer, exchange, and use of health data and expertise across borders. Most interviews with informants in the capitals (Phnom Penh and Hanoi) were conducted in English, while the majority of meetings at the provincial level were in Cambodian or Vietnamese and simultaneously translated into English by local research partners. Depending on the context and consent, interviews were either recorded (and then subsequently translated into English, where needed) or extensive notes were taken during the meetings. All interviews were conducted face-to-face by ML, alone or together with either ST or LMS. Relevant documents (such as reports and published papers) were also collected and reviewed at different stages to clarify points or triangulate information.

The analysis of qualitative data was structured around three categories mapped across the dataset, representing different yet interlinked elements in data and information sharing processes: (1) the nature of shared data and information; (2) the nature of communication channels; and (3) how information flow may be affected by the local, regional, and global system of rules and arrangements. Within each category, emerging themes were identified through an iterative process of categorisation and constant comparison of findings from the interview transcripts [40]. Collected material was organized and coded using QSR nVivo 10 software package to manage the complexity of information. Preliminary findings were presented at two international workshops in Cambodia and Vietnam, both held in March 2017, where feedback from stakeholders was obtained and provided additional insights into the process of analysis.

## Results

Overall, we held 60 meetings in Cambodia (*n* = 28) and Vietnam (*n* = 32), where domestic or international stakeholders were invited to discuss their views on the given topic and respond to our questions (Table 2). In Cambodia, we had 10 meetings at the central level (including directors and staff of health departments and vertical programmes as well as data managers), 10 meetings at provincial health departments (including directors, deputy directors, and data managers), and 8 meetings with representatives of international organisations or non-governmental organisations (NGOs) in health or other sectors. In Vietnam, we had meetings at 10 central institutions within the Ministry of Health (including directors of public health institutes, health departments, and hospital directors), 18 meetings at provincial government departments, and 4 meetings at international organisations. Interview meetings were attended by individual participants or a small group of colleagues (from 2 to 6) working in the same office or organisation. In the presentation of findings, structured around the three overarching categories noted earlier, key points and anonymised citations are referenced by the unique identifiers CAM*-n* and VIET*-n* for respondents in Cambodia and Vietnam respectively.

**Table 2:** Interviews by country and type of organisation

	Cambodia	Vietnam
Government (central level)	10	10
Government (provincial level)	10	18
International organisations/NGOs	8	4
Total	28	32

### The nature of shared data and information

The majority of participants had positive views and experiences about their involvement in regional initiatives; reasons that were frequently mentioned to explain the value of regular and sustained communications between country partners included alert in the event of emergency, better understanding of disease epidemiology, and exchange of good practices and experiences. Malaria experts further noted that international case tracking is particularly important in the current context of regional strategy towards malaria elimination, given the cross-border dimension of residual malaria transmission and the need for detailed information about malaria cases traveling across countries (CAM-22, CAM-26). However, challenges emerged in relation to the nature and reliability of shared data, as illustrated in the sections below.

#### Data comparability

One important area of concern was the extent to which shared data from different countries can be used to produce a regional mapping of disease burden, given gaps in national information systems and imbalances in their ability to provide an estimate of the true disease incidence. As one international expert in Cambodia noted, the comparison of raw data could lead to wrong epidemiological conclusions and potentially affect resource allocation in biased ways:

*“If one country is saying they have 30 cases and another country 50… it seems that the problem in the second country is worse than in the first country, but it may just be that the other country was not able to detect many cases…”* (CAM-20).

Two informants at the Ministry of Health in Cambodia further highlighted a conflict between a single reporting system for cross-border activities and the fragmentation of the disease information systems in the country (CAM-14, CAM-17). Similarly, one malaria epidemiologist remarked:

*"What is Cambodia sharing? Data from the village malaria workers programme that go to the CNM* [the National Malaria Centre] *or data from the NHIS* [the National Health Information System]*… the two systems are not well integrated…"* (CAM-29).

#### Inconsistencies in case definitions

The use of differing case definitions and formats in data collection and reporting was identified as another barrier to data comparability (VIET19, VIET26). Some informants recognised it would be useful to have a regional analysis of imported malaria or dengue cases since this would enable better understanding of the role of population mobility in disease transmission. However, one data manager at the MoH in Cambodia pointed out that comparative analysis of such data is problematic due to inconsistencies in the definition of imported cases across countries in the region, depending on whether internal or international mobility is seen as the main driver of disease transmission and other variables. Similar issues were noted by HIV experts in Vietnam:

*“There is no standard methodology for at risk populations, like IDU* [injecting drug users]*, MSM* [men who have sex with men] *and sex workers… many methods have been tried… but there are no regional guidelines… there have been many pilot studies, using different approaches… but then it is difficult to integrate different datasets and not really cost-effective…”* (VIET19).

The case of the regional malaria database further illustrates challenges resulting from discrepancies in data collection methods and formats. As part of the regional strategy for malaria elimination in the Greater Mekong Subregion, national stakeholders and international actors agreed to develop a regional database with data on malaria cases from all countries in the region. However, discrepancies in data collection systems emerged as a major barrier to project implementation, requiring a laborious process of negotiations on common indicators. Two informants in Cambodia explained:

*“We had several meetings to establish a regional database… But the main problem is the type of data… that are very different in each country… so at the meeting, we decided to share basic indicators… Another problem is the unit of data collection, which can vary considerably from township, to provinces to operational districts, to communes…”* (CAM-22).

*“We are developing a web-based regional database. It took about three days to get countries to agree on indicators… whether it should be at the commune level, at the provincial level, or at the country level (…) eventually we agreed on confirmed cases disaggregated by species, number of person tested, depending on the capacities; admitted malaria cases, including data from the private sector if available; severe malaria cases; malaria deaths; and completeness of health facility report”* (CAM-24).

The second informant also noted that completeness of reporting – defined as the proportion of health facilities that are actually reporting out of the total proportion of facilities that are expected to report – is a key variable in the development of the regional database: “this point is very important as missing data can be incorporated into the modelling exercise so that we can have an estimate of real figures” (CAM-24).

#### Reliability of information systems and trustworthiness of shared information

Another area of concern was that perceptions about the reliability of the information source can affect the credibility of international communications. One health sector manager at the central level in Cambodia, who was involved in several regional initiatives, explained that “district hospital in some countries can do many testing…. when they report pneumonia, it is really pneumonia… they tell everything; but when we report cases, it is only based on clinical symptoms… they say it is not reliable” (CAM-18).

Further, a few participants noted that asymmetries in capacities to detect outbreaks may result in different risk perceptions, undermining the potential for collective action. While informants in both Cambodia and Vietnam reported examples of joint outbreak investigations conducted in recent years, one quarantine officer in Vietnam explained:

“In 2013, we had some suspected cases of [cross-border] cholera and we wanted to do a joint outbreak investigation with Cambodia but at that time most suspected cases were identified only in Vietnam so the priorities were different.” (VIET-01).

### Communication channels and information flows

Over the past two decades, channels for cross-border sharing of data, information, and expertise in Southeast Asia have multiplied as well as the frequency of international communications. In addition to routine exchange of epidemiological reports (usually by email or shared database), the establishment of bilateral and multilateral agreements for infectious disease control has promoted an intensification in cross-border and regional meetings, where officers from different countries present and share data, information, and expertise in their area of responsibility (usually by slide presentation). Representation at cross-border meetings is variable, ranging from small gatherings between local health authorities to large inter-sectoral meetings involving a delegation of about 40 people or more from each country. One informant in Cambodia stressed that broad representation and the involvement of provincial governors are crucial to promote inter-sectoral cooperation and high-level stewardship (CAM-04). Further, meetings were seen to provide an opportunity to initiate or consolidate personal relations between health professionals in different countries, which can be used to promote informal communications in the event of disease outbreaks or other public health needs (CAM-10).

#### The politics of participation at cross-border and regional meetings

Despite positive views about the value of regional meetings, some narratives illustrate that meetings can be a locus where imbalances in capacities are reflected, influencing the dynamics of data and information sharing in undesirable ways. In particular, two international experts reported that different levels of technical proficiency and ownership of data and data collection systems impacted on the ability of country partners to share data and speak confidently:

*“When a country owns and is confident of their data…. They are able to defend it… they are more willing to share… but when you don’t build the capacity of a country to build and own an information system…there is a problem… Vietnam data come monthly… they have Global Fund support but full data ownership, so they feel more confident…”* (CAM-24)*.*

*“In my previous job, I often attended regional meetings (…) I noticed that countries like Thailand and China speak confidently about their information system, but* [representatives of] *countries that have weaknesses in information systems say much less…”* (CAM-20).

In addition, a number of informants, particularly in Cambodia, noted that gaps in capacities to finance bilateral cooperation resulted in imbalances in decision-making power, including the power to set the meeting agenda:

*“We would like to invite partners from other countries* [to attend meetings in Cambodia] but *we no longer have budget for that. So, we are invited* [by neighbouring countries] *and cannot decide* [the content and schedule of the meeting]*”* (CAM-08).

This issue was effectively summarised by a senior health manager in Cambodia, who noted with some frustration that “Cambodia always participate, participate, participate…” (CAM-18).

#### Information flow and data collection systems

Challenges to the flow of data within and across countries were also identified in relation to technical capacities and gaps in the information technology (IT) infrastructure. Despite improvements in IT and network access, in both Cambodia and Vietnam routine surveillance data are still collected on paper forms in many health facilities, particularly at the community level. Thus, the availability of data in electronic format for dissemination requires a lengthy process of data entry, which may prevent the timely circulation of information, both at the national and international level. As one data manager in Cambodia explained:

*“Most data are still collected on paper forms, although we are developing an online reporting system, funded by the World Bank. This will take about two years (…) For the moment, reporting is time consuming because the supervisors at the district level must visit all health centres in their catchment area to collect information every month. The supervisor at the provincial level collects and checks data from all operational districts every two months. The national supervisor then visits each provincial health department quarterly to get the provincial report”* (CAM-11).

Another informant in Cambodia explained:

*“Sometimes our reporting is not timely and complete – especially because it is difficult to have all data from other programs (…) as they are under other staff, and sometimes the national programme for malaria or HIV do not have all data every week”* (CAM-16).

#### Language

Many participants pointed out that language was an important variable affecting the exchange of information and access to shared data resources. Communications are relatively straightforward between health professionals from Laos and Thailand (as Lao and Thai are mutually understandable) or in bilingual areas along the borders (such as Surin province in Thailand, where residents can often speak both Cambodian and Thai). Further, central level managers in regional countries have usually a good command of the English language, which is used as the lingua franca in most international communications in Southeast Asia. In other contexts, however, language was identified a major barrier to cross-border cooperation or the use of regional data (CAM-06, CAM-14, CAM-21), as the following excerpts illustrate:

*“Email exchange are in English… but there are language limitations so we cannot explain well the full situation. Due to language problems, it is difficult to explain effectively… even if we have translators during the meetings…”* (VIET-17).

*“In the past, the regional website was only available in English, so many people could not access the information well. Now we use it more because it has recently been translated in Vietnamese”* (VIET16).

### Institutional and organisational arrangements

In addition to the nature of shared data and communication channels, participants discussed points related to the wider context of institutional arrangements, rules, and contexts which are in place to support data and information sharing.

#### National regulations and norms

The analysis of interviews showed that local rules and arrangements shaped the ability of local stakeholders to share data in variable ways, resulting in potential asymmetries in data exchange between partners even when informal agreements exist to promote international communications. In Cambodia, the government has introduced a number of reforms towards increasing devolution of authority and decision-making power to provincial health departments [41]. As a result, local authorities have reportedly more autonomy to manage health cooperation with provinces in neighbouring countries, including sharing of data and information. In Vietnam, there have also been efforts to devolve the management of the health sector [42]. However, health system governance remains fairly centralised with limited decision-making discretion at the provincial level, particularly on international issues. As reported by several informants, this norm may enhance high-level coordination and national stewardship in the event of emergencies, but constrains the ability of local managers to engage in cross-border collaborations:

*“If we need information from other countries, it is always the central level - because this is government… I think they work with the WHO and other countries… If we need information from other countries, they will do…”* (VIET05).

#### Regional governance

In both countries, there was a consensus amongst participants that the IHR 2005 were an effective instrument to mandate the global notification of health emergencies of international concern; however, the lack of a clear legal framework to support regional programmes (usually based on “soft law” agreements such as memoranda of understanding) was seen as a barrier to the establishment of direct communication across borders. As one informant pointed out, “the problem is there is no legal framework (…) we feel strange, we don’t do it [contact colleagues in neighbouring countries], we cannot do it…” (VIET-05). Similarly, another informant in Vietnam stressed that the lack of agreed standard operating procedures was a barrier to the implementation of joint responses to cross-border disease outbreaks (VIET-26). On another note, one informant in Cambodia lamented the proliferation of bilateral and multilateral public health agreements, suggesting that ASEAN should be the overarching framework to coordinate regional public health initiatives (CAM-08).

#### Sustainability of donor-funded initiatives

Financing arrangements and the sustainability of public health programmes emerged as another important structural issue affecting dynamics of regional data sharing. Over the past two decades, the development and maintenance of regional public health networks has been financed predominantly by non-regional donors through various forms of “triangular” cooperation, an arrangement in which one or more donors provide financial support and technical input to promote South-South cooperation [43]. However, donor funding for regional initiatives has decreased recently due to changing donor priorities and expectations that participating countries would sustain cross-border partnerships with their own resources. In our interviews, we found that more resourced regional partners, particularly Thailand and, to a lesser extent, Vietnam, have provided increasing support to neighbouring countries (CAM-02, VIET-30, VIET-32), especially in cross-border areas where population movement is seen as a major driver of disease transmission, such as the Thailand-Myanmar border. However, South-South financing has tended to prioritise training and capacity building, and has not been able to match the level of funding from international donors. Thus, data and information sharing activities reportedly discontinued in some contexts or reduced, as noted by one senior officer in Vietnam (VIET-29) and two officers at provincial health departments in Cambodia:

*“We were given a satellite phone as part of a surveillance programme a few years ago to use it for international communication with our partners. We still have the phone, but no funding to use it and it is more expensive than a normal phone (…)”* (CAM-21).

*“In the past they* [the programme] *also provided funds for local staff, but now they give only a small contribution to pay internet expenses… about US $10-15 a month… it’s not enough”* (CAM-01)*.*

## Discussion

This study aimed to elicit stakeholders’ perspectives on the range of issues affecting health data and information sharing across borders for infectious disease surveillance. Many informants recognised the value of regional public health networks and reported examples of positive outcomes and achievements, but different barriers to data sharing were identified. While the public health literature does highlight an obvious need for more and timely exchange of epidemiological information to address health threats of international concern, our findings document that the movement of data and information from production sites to other places may be challenging. Specific issues that were seen to affect “data journeys” [44] across the three dimensions in our investigations include imbalances in capacities of national health systems to provide reliable information on disease burden and outbreaks, different data collection and reporting systems, language barriers, differing national structures and rules that govern the circulation of health information inside and outside the country, and the sustainability of financing arrangements.

Some of these issues have been noted or explored in the literature. For example, the effects of source credibility, including expertise and trustworthiness, have been examined in studies of health communication as well as the influence of different communication channels and culture on message delivery and effectiveness [45]. In addition, the problem of comparability of data from different sources has been recognised and addressed in epidemiology [46]. Yet the examination of these tensions “in vivo”, from the perspective of health authorities and practitioners, is a novel contribution of this study, which enables a better understanding of challenges to data sharing and how these may affect the practical work of regional public health networks. As the analysis of interviews indicates, perceptions about the reliability of data and information sources may affect international cooperation not only for routine surveillance and monitoring purposes, but can also stifle the potential for collective action in the event of emergencies. Furthermore, awareness of technical proficiency and ownership of data and data collection systems were perceived to provide a comparative advantage in social interactions at regional meetings and the ability of stakeholders to engage in regional collaborations. Taken together, these points highlight the socio-technical nature of data and information sharing - a complex social process shaped by linkages between capacities, risk perceptions, trust, and behaviour.

Another original contribution of this study is a deeper understanding of these issues in the context of South-South cooperation. While the literature has highlighted issues of fairness in South-North partnerships for data sharing [47], articulated concerns about donor involvement in regional surveillance networks [48], and examined the problem of sustainability of donor-sponsored regional programmes [49], less attention has been paid to South-South data sharing patterns and, more broadly, collaborative dynamics between regional partners. In recent years, there has been a strong consensus on the value of South-South cooperation and its potential to foster more sustainable pathways to development, encourage knowledge transfer that is better suited to local contexts, and other positive externalities such as enhanced sense of ownership, self-reliance and independence [50, 51]. Findings from this study provide no insights to dispute these claims; yet, we found that South-South collaborations may also reflect asymmetries in technical capacities and decision-making. Despite an emphasis on mutual learning and equal participation, the content and flows of information may be directed by dominant country partners as well as those of donors involved in triangular forms of cooperation. While these issues have been explored in critical studies of international development [52, 53], little research has focused on their practical implications for public health and knowledge transfer in LMICs. Our study highlights this is an important issue that requires further attention if we are to better understand ways to establish mechanisms for equitable cooperation - in the health sector and beyond.

Further reflecting on our findings, we can provide additional insights which can be useful to inform policy and planning in this area. As we have seen, data and information sharing across borders is a complex and demanding task requiring convergence and communication between systems that are embedded in local contexts and structures. While global standards, guidelines, and rules have been developed to facilitate information sharing, comparative analyses, and global mapping of disease burden [54], states remain the main framework for the organisation of societies and their institutions, including the health sector. As a result, health information systems are variably shaped by national structures, capacities, rules, and differing approaches to data collection, validation, reporting, and dissemination. Given these differences, the establishment of regional public health systems requires considerable efforts to harmonise different practices and standards, iron out discrepancies, and create a common platform which can promote equitable exchange and fruitful use of shared data.

What can be done to address these challenges? Recent studies in network analysis on the role of third-party brokers as enablers of inter-sectoral cooperation offer useful insights [55]. In particular, Collins-Dogrul provides convincing evidence and arguments that broker organisations were crucial to initiate and maintain cooperation between health authorities at the USA-Mexico border, countering the centrifugal forces of national structures, interests, and allegiances [56]. Similarly, our study highlights the need for brokering at different levels in the data sharing process: “epistemic brokering” [57] in the form of data curation, validation, and modelling is required to make the travel of data across borders fruitful; “cultural brokering” [58] is needed to mediate linguistic barriers and, at the same time, promote a shared discursive frame about regional public good which can value and legitimise cooperation; “regulatory brokering” is also necessary to create a common legal framework for cross-border data sharing activities, including provisions to address ethical issues that may result from the sharing of patient records. Importantly, the involvement of a third-party organisation has the potential to redress imbalances between partners by distributing pooled resources and responsibilities in a more equitable way, a role that may be facilitated by the perceived aura of neutrality that brokers enjoy. To be sure, broker organisations have their own interests and can even reinforce relational inequalities, if a normative orientation towards equal participation is not in place. However, as Collins-Dogrul put it, to retain their position, brokers must “use their authority and influence to create, sustain, and enhance connections between people and organizations in ways that are perceived as improving outcomes for many members of the network, not just the broker” (p. 996) [59].

In Southeast Asia, past experiences in regional cooperation illustrate significant involvement of different types of third party brokers in public health programmes, including coordinating offices of disease surveillance networks, the WHO, and regional organisations such as the Association of Southeast Asian Nations (ASEAN). An evaluation of the brokering role of these organisations in support of data and information sharing networks was beyond the scope of this research project. Nonetheless, we should note that ASEAN is currently in a good position to play a more proactive role in regional health affairs and act as a catalyst for the diverse range of initiatives and programmes. In recent years, ASEAN has been the focal point in a number of regional health programmes [22, 37], but its institutional potential has not been fulfilled, due to limited financial resources and a weak legal framework based on the rule of consensus [60]. However, the ongoing progress towards the establishment of the ASEAN Economic Community is an important upgrade in regional cooperation which will require increasing regulatory convergence on several trade issues, with potential spill-over effects in public health policy, similar to developments in the European Union [61]. Currently, as part of the ASEAN work programme in the health sector (2016–2020), the ASEAN Secretariat is mandated to coordinate a wide range of activities for the prevention and control of infectious diseases, including continued support to existing disease surveillance networks, preparedness through joint simulation exercises and the ASEAN Field Epidemiology Training Programme (FETP), led by Thailand [62]. Stronger ASEAN involvement in these areas would be useful to enhance regional ownership of shared datasets and sustainability, since the ASEAN budget can be supported by donor contributions but is not entirely dependent on the volatility of foreign aid assistance. In addition, the creation of a regional public health centre under the ASEAN framework could serve the key role of clearinghouse and central data management unit, improving usability, reliability, and wide dissemination of shared data inside and outside the region. Efforts to achieve this would need to align with global governance mechanisms and organisations, particularly the WHO, to promote wider integration and use of regional datasets as well as global stewardship in the event of emergencies which transcend national and regional borders.

### Study limitations and suggestions for further research

This study Is based on a unique collection of stakeholders’ views about public health data and information sharing across borders in a developing context. To the best of our knowledge, this is the first study of this kind and, hopefully, our analysis can be useful to inform and encourage further research on these issues. We should note, however, several study limitations. First, key informant interviews are particularly vulnerable to social desirability biases, especially when informants represent institutions and government departments. In our study, whenever possible, we tried to encourage personal and critical engagement with the given topics. At times, however, this was difficult to achieve, particularly in group interviews at government departments, where meetings were more formal and provided less opportunities for questioning and probing. Second, given the exploratory nature of this study, we could generate insights on the socio-technical complexity of health data and information sharing, but in-depth exploration of emerging issues would require additional work and specific methodologies. For example, network analysis could be used to chart structural patterns in the flow of data and information within and across countries, including centrality of nodes, reciprocity in information exchange, clustering, and, importantly, brokerage dynamics. In addition, participant observation at regional meetings, informed by interactionist perspectives [63], would provide a deeper understanding of ways in which imbalances in access to resources may affect, in practice, knowledge exchange between country partners. Lastly, this study was conducted in a particular geopolitical and public health context of regional cooperation. As noted earlier, the focus on Southeast Asia provides strategic opportunities to explore different aspects of the phenomenon investigated here. However, future research work could be conducted in other areas to enable comparative case study analysis and provide stronger evidential bases for theory development and generalisation.

## Conclusions

Our study documents ways in which imbalances between national health systems and capacities may affect the practice of cross-border data and information sharing, suggesting that best practices require significant involvement of an independent third-party brokering organisation or office, which can redress gaps between country partners at different levels in the data sharing process, create meaningful communication channels and make the most of shared information and data sets. By way of conclusion, we can extend this argument a step further and speculate that data and information sharing works better if supported by strong multilateral arrangements, since these typically involve a third–party organisation with responsibilities for budget administration, data management, and overall coordination. By contrast, a bilateral cooperation agreement is likely to have a weaker brokering orientation and is generally thought to be a less effective mechanisms to address collective good problems, as documented in studies of aid relationships [64]; this is even more apparent in contexts where transnational issues have a wide regional dimension and require harmonisation, coordination, and integration between more than two parties. Further attention to these issues is much needed in the present historical context. In the past few years, political developments in Europe and the United States have coincided with a crisis of multilateralism and regional cooperation - two foundations of the global order from the postwar to the present [65]. Thus, research that can inform a better understanding of ways in which these changes may affect cooperation amongst countries and the achievement of the common good, in public health as in other policy areas, is timely.

## References

[1] UNOCHA. Strengthening border surveillance between Ebola affected countries. Ebola Bulletin. United Nations Organization for the Coordination of Humanitarian Affairs. 2015.

[2] Coltart Cordelia E. M., Lindsey Benjamin, Ghinai Isaac, Johnson Anne M., Heymann David L. (2017). The Ebola outbreak, 2013–2016: old lessons for new epidemics. Philosophical Transactions of the Royal Society B: Biological Sciences.

[3] Imwong M, Suwannasin K, Kunasol C, Sutawong K, Mayxay M, Rekol H, Smithuis FM, Hlaing TM, Tun KM, van der Pluijm RW (2017). The spread of artemisinin-resistant plasmodium falciparum in the greater Mekong subregion: a molecular epidemiology observational study. Lancet Infect Dis.

[4] Ashley EA, Dhorda M, Fairhurst RM, Amaratunga C, Lim P, Suon S, Sreng S, Anderson JM, Mao S, Sam B (2014). Spread of artemisinin resistance in plasmodium falciparum malaria. N Engl J Med.

[5] Bwire G, Mwesawina M, Baluku Y, Kanyanda SS, Orach CG (2016). Cross-border cholera outbreaks in sub-Saharan Africa, the mystery behind the silent illness: what needs to be done?. PLoS One.

[6] Bogoch II, Brady OJ, Kraemer MUG, German M, Creatore MI, Kulkarni MA, Brownstein JS, Mekaru SR, Hay SI, Groot E (2016). Anticipating the international spread of Zika virus from Brazil. Lancet.

[7] Pfeiffer DU, Otte MJ, Roland-Holst D, Inui K, Nguyen T, Zilberman D (2011). Implications of global and regional patterns of highly pathogenic avian influenza virus H5N1 clades for risk management. Vet J.

[8] Gustavsen K, Sodahlon Y, Bush S (2016). Cross-border collaboration for neglected tropical disease efforts-lessons learned from onchocerciasis control and elimination in the Mano River union (West Africa). Glob Health.

[9] Tarantola Arnaud, Goutard Flavie, Newton Paul, de Lamballerie Xavier, Lortholary Olivier, Cappelle Julien, Buchy Philippe (2014). Estimating the Burden of Japanese Encephalitis Virus and Other Encephalitides in Countries of the Mekong Region. PLoS Neglected Tropical Diseases.

[10] Pisani E, AbouZahr C (2010). Sharing health data: good intentions are not enough. Bull World Health Organ.

[11] WHO (2007). Cross-border collaboration on emerging infectious diseases. Report of the bi-regional meeting Bangkok, 26-28 February 2007.

[12] Chatham House. A Guide to sharing the data and benefits of public health surveillance. Centre on global health security. London: Chatham House; 2017.

[13] Liverani M, Coker R (2012). Protecting Europe from diseases: from the international sanitary conferences to the ECDC. J Health Polit Policy Law.

[14] Ope Maurice, Sonoiya Stanley, Kariuki James, Mboera Leonard E.G, Gandham Ramana N.V, Schneidman Miriam, Kimura Mwihaki (2013). Regional Initiatives in Support of Surveillance in East Africa: The East Africa Integrated Disease Surveillance Network (EAIDSNet) Experience. Emerging Health Threats Journal.

[15] Phommasack Bounlay, Jiraphongsa Chuleeporn, Ko Oo Moe, Bond Katherine C., Phaholyothin Natalie, Suphanchaimat Rapeepong, Ungchusak Kumnuan, Macfarlane Sarah B. (2013). Mekong Basin Disease Surveillance (MBDS): A Trust-Based Network. Emerging Health Threats Journal.

[16] Lover AA, Gosling R, Feachem R, Tulloch J (2016). Eliminate now: seven critical actions required to accelerate elimination of plasmodium falciparum malaria in the greater Mekong subregion. Malar J.

[17] Liverani M, Hanvoravongchai P, Coker RJ (2012). Communicable diseases and governance: a tale of two regions. Glob Public Health.

[18] Leventhal Alex, Ramlawi Assad, Belbiesi Adel, Sheikh Sami, Haddadin Akhtam, Husseini Sari, Abdeen Ziad, Cohen Dani (2013). Enhanced Surveillance for Detection and Management of Infectious Diseases: Regional Collaboration in the Middle East. Emerging Health Threats Journal.

[19] Leventhal A, Ramlawi A, Belbiesi A, Balicer RD (2006). Regional collaboration in the Middle East to deal with H5N1 avian flu. BMJ.

[20] Bruniera-Oliveira R, Horta MAP, Varan A, Montiel S, Carmo EH, Waterman SH, Verani JFS. Epidemiological surveillance of land borders in North and South America: a case study. Rev Inst Med Trop Sao Paulo. 2017;59:e68. 10.1590/S1678-9946201759068 PMC5679680 29116288

[21] UNDP (2007). Evaluation of UNDP contribution to south-south cooperation.

[22] Curley M, Thomas N (2004). Human security and public health in Southeast Asia: the SARS outbreak. Aust J Int Aff.

[23] Thomas N (2006). The regionalization of avian influenza in East Asia: responding to the next pandemic(?). Asian Surv.

[24] Yu-Hung Lai A, Kamradt-Scott A, Coker R. The coming pandemic: transnational health challenges in East and Southeast Asia. In: Tan, ATH, editor. East and South-East Asia: international relations and security perspectives. New York: Routledge; 2013.

[25] Hameiri S (2014). Avian influenza, ‘viral sovereignty’, and the politics of health security in Indonesia. Pac Rev.

[26] Ross E (2014). Perspectives on data sharing in disease surveillance.

[27] van Panhuis WG, Paul P, Emerson C, Grefenstette J, Wilder R, Herbst AJ, Heymann D, Burke DS (2014). A systematic review of barriers to data sharing in public health. BMC Public Health.

[28] Jao I, Kombe F, Mwalukore S, Bull S, Parker M, Kamuya D, Molyneux S, Marsh V (2015). Research stakeholders' views on benefits and challenges for public health research data sharing in Kenya: the importance of trust and social relations. PLoS One.

[29] Denny SG, Silaigwana B, Wassenaar D, Bull S, Parker M (2015). Developing ethical practices for public health research data sharing in South Africa: the views and experiences from a diverse sample of research stakeholders. J Empir Res Hum Res Ethics.

[30] Hate K, Meherally S, Shah More N, Jayaraman A, Bull S, Parker M, Osrin D (2015). Sweat, skepticism, and uncharted territory: a qualitative study of opinions on data sharing among public health researchers and research participants in Mumbai, India. J Empir Res Hum Res Ethics.

[31] Yin R (2014). Case study research: design and methods.

[32] Coker RJ, Hunter BM, Rudge JW, Liverani M, Hanvoravongchai P (2011). Emerging infectious diseases in Southeast Asia: regional challenges to control. Lancet.

[33] Smith Gueye C, Newby G, Hwang J, Phillips AA, Whittaker M, MacArthur JR, Gosling RD, Feachem RG (2014). The challenge of artemisinin resistance can only be met by eliminating plasmodium falciparum malaria across the greater Mekong subregion. Malar J.

[34] Sharma M, Chatterjee A (2012). Partnering with law enforcement to deliver good public health: the experience of the HIV/AIDS Asia regional program. Harm Reduct J.

[35] ADB (2014). Prevention and control of avian influenza in Asia and the Pacific. Project report.

[36] WHO (2013). Emergency response to artemisinin resistance in the greater Mekong subregion: regional framework for action 2013–2015.

[37] Liverani M, Hanvoravongchai P, Coker R (2013). Regional mechanisms of communicable disease control in Asia and Europe.

[38] Socheat D, Denis MB, Fandeur T, Zhang Z, Yang H, Xu J, Zhou X, Phompida S, Phetsouvanh R, Lwin S (2003). Mekong malaria. II. Update of malaria, multi-drug resistance and economic development in the Mekong region of Southeast Asia. Southeast Asian J Trop Med Public Health.

[39] Reddy A, Htin KC, Shwe YY (2012). HIV and AIDS data hub for Asia Pacific: a regional tool to support strategic information needs. Western Pac Surveill Response J.

[40] Strauss A, Corbin J (1998). Basics of qualitative research.

[41] Annear P, Jacobs B, Nachtnebel M (2015). The Kingdom of Cambodia. Health system review.

[42] Ramesh M (2013). Health care reform in Vietnam: chasing shadows. J Contemp Asia.

[43] Rhee H, Kharas H, Makino K, Jung W (2011). Promoting South-South cooperation through knowledge exchange. Catalyzing Development.

[44] Leonelli S (2016). Data-centric biology. A philosophical study.

[45] Kreuter MW, McClure SM (2004). The role of culture in health communication. Annu Rev Public Health.

[46] Gibbons CL, Mangen MJ, Plass D, Havelaar AH, Brooke RJ, Kramarz P, Peterson KL, Stuurman AL, Cassini A, Fèvre AN, Kretzschmar MEE. Measuring underreporting and under-ascertainment in infectious disease datasets: a comparison of methods. BMC Public Health. 2014;14:147. 10.1186/1471-2458-14-147 PMC4015559 24517715

[47] Tangcharoensathien V, Boonperm J, Jongudomsuk P (2010). Sharing health data: developing country perspectives. Bull World Health Organ.

[48] Calain P (2007). From the field side of the binoculars: a different view on global public health surveillance. Health Policy Plan.

[49] Moore M, Dausey D, Phommasack B, Touch S, Lu G, Lwin Nyein S, Ungchusak K, Vung N, Oo M. Sustainability of sub-regional disease surveillance networks. Global Health Gov. 2012;5(2):1-43.

[50] UN (2012). Framework of operational guidelines on United Nations support to South-South and triangular cooperation. High-level Committee on South-South Cooperation. Seventeenth session. New York, 22–25 May 2012.

[51] OECD (2011). Unlocking the potential of south-south cooperation.

[52] McEwan C, Mawdsley E (2012). Trilateral development cooperation: power and politics in emerging aid relationships. Dev Chang.

[53] Chandy Laurence, Kharas Homi (2011). Why Can't We All Just Get Along? The Practical Limits to International Development Cooperation. Journal of International Development.

[54] WHO (2017). WHO methods and data sources for global burden of disease estimates 2000–2015.

[55] Obstfeld D (2005). Social networks, the tertius iungens orientation, and involvement in innovation. Adm Sci Q.

[56] Collins-Dogrul J (2006). Managing US-Mexico "border health": an organizational field approach. Soc Sci Med.

[57] Raymond CW (2014). Conveying information in the interpreter-mediated medical visit: the case of epistemic brokering. Patient Educ Couns.

[58] Johnson J (2004). The emergence, maintenance, and dissolution of structural hole brokerage within consortia. Commun Theory.

[59] Collins-Dogrul J (2011). Tertius iungens brokerage and transnational intersectoral cooperation. Organ Stud.

[60] Acharya Amitav (2014). Constructing a Security Community in Southeast Asia.

[61] Greer S (2006). Uninvited Europeanization: neofunctionalism and the EU in health policy. J Eur Public Policy.

[62] ASEAN (2016). Working together to address complex health challenges.

[63] Goffman E (1967). Interaction ritual: essays on face-to-face behavior.

[64] Gulrajani N (2016). Bilateral versus multilateral aid channels. Strategic choices for donors.

[65] Lazarou E (2017). The future of multilateralism crisis or opportunity?.

